# The Prevention of Cholera Infantum and Kindred Diseases

**Published:** 1887-09

**Authors:** Victor C. Vaughan

**Affiliations:** Professor of Physiological Chemistry in the University of Michigan and Member of the State Board of Health


### The Prevention of Cholera Infantum and Kindred Diseases.

BY VICTOR C. VAUGHAN, M.D., PH.D.,
Professor of Physiological Chemistry in the University of Michigan and Member of the State Board of Health.

(Abstract of a Paper on the Chemistry of Tyrotoxicon, its Action upon Lower Animals, and its Relation to the Summer Diarrhœas of Infancy.)

The experiments upon animals (which are given in the full paper to be published in the annual report of the State Board of Health) convince me that the development of tyrotoxicon in milk is a frequent cause of cholera infantum and kindred affections. When we remember that these diseases are most prevalent among the poorer classes of our large cities, where fresh milk is almost unknown, we can readily understand their frequency. By such people milk is often not obtained until it has begun to sour; then it is kept at a high temperature, and often in a most foul atmosphere, and we all know something of the readiness with which milk takes up bad odors. This milk is then eaten by the little ones, who are weakened by poverty and every thing that poverty means, insufficient food generally, and that of the poorest quality, insufficient clothing, insufficient and vitiated air. With these facts before us, it is not surprising that in all our large cities thousands of our children die annually from the summer diarrhœas. Moreover, in our country places how little attention is given to the food of children we all know from actual observation. Cows stand and are milked in filthy barns and yards. The udders are generally, so far as my observation goes, not washed before the milking; the vessels for the milk are frequently found not as clean as they should be. Then there are the thousands of children that must draw their sustenance from bottles, the cleansing of which is in many families not properly attended to. Crusts of decomposing milk form around the neck of the bottle, in the tube and nipple, and lead to rapid decomposition of the entire contents of the bottles. I think that one of the most important advantages to be secured to breast-fed children arises from the lessened danger of infection of the milk with germs which may produce poisonous ptomaines.

I would not claim that decomposed milk is the sole cause of the summer diarrhœas of children, nor would I claim that tyrotoxicon is the only poison that may be developed in milk ; it is only one of a large class of bodies which are produced by putrefaction, and many of these are cathartic in action.

RULES FOR THE PREVENTION OF THE DEVELOPMENT OF TYROTOXICON IN MILK.

1. The cows should be healthy, and the milk of any animal which seems indisposed should not be mixed with that from the perfectly healthy animals.

2. Cows must not be fed upon swill, or the refuse of breweries or glucose factories, or any other fermented food.

3. Cows must not be allowed to drink stagnant water, but must have free access to pure, fresh water.

4. Cows must not be heated or worried before being milked.

5. The pasture must be free from noxious weeds, and the barn and yard must be kept clean.

6. The udders should be washed, if at all dirty, before the milking.

7. The milk must be at once thoroughly cooled. This is best done by placing the milk can in a tank of cold spring or ice-water, the water being of the same depth as the milk in the can. It would be well if the water in the tank could be kept flowing; indeed, this will be necessary unless ice-water is used. The tank should be thoroughly cleaned every day to prevent bad odors. The can should remain uncovered during the cooling and the milk should be gently stirred. The temperature should be reduced to 60° F. within an hour. The can should remain in the cold water until ready for delivery.

8. In summer, when ready for delivery, the top should be placed on the can and a cloth wet in cold water should be spread over the can, or refrigerator cans may be used. At no season should the milk be frozen; but no buyer should receive milk which has a temperature higher than 65° F.

9. After the milk has been received by the consumer, it should be kept in a perfectly clean place, free from dust, at a temperature not exceeding 60° F. Milk should not be allowed to stand uncovered, even for a short time in sleeping or living rooms. In many of the better houses in the country and villages, and occasionally in the cities, the drain from the refrigerator leads into a cesspool or kitchen drain. This is highly dangerous; there should be no connection between the refrigerator and any receptacle of filth.

10. The only vessels in which milk should be kept are tin, glass, or porcelain. After using the vessel it should be scalded, and then, if possible, exposed to the air.

